# Detection of BCR-ABL T315I mutation by peptide nucleic acid directed PCR clamping and by peptide nucleic acid FISH

**DOI:** 10.1186/s40364-015-0039-y

**Published:** 2015-07-03

**Authors:** Valentina Rosso, Enrico Bracco, Roberto Pedrola, Sonia Carturan, Elisabetta Signorino, Jessica Petiti, Chiara Calabrese, Paolo Nicoli, Marco De Gobbi, Valentina Gaidano, Daniela Gallo, Stefano Ulisciani, Carmen Fava, Giovanna Rege-Cambrin, Francesco Frassoni, Giuseppe Saglio, Daniela Cilloni

**Affiliations:** Department of Clinical and Biological Sciences, University of Turin, Turin, Italy; Department of Oncology, University of Turin, Turin, Italy; Department of Pediatric Hemato-Oncology and Stem Cell and Cellular Therapy Laboratory, Institute G. Gaslini, Largo G Gaslini, Genoa, 16147 Italy

**Keywords:** BCR-ABL1, T315I mutation, Chronic Myeloid Leukemia, PNA

## Abstract

**Background:**

Mutations of the *BCR-ABL1* fusion gene represent a well established cause of resistance to tyrosine kinase inhibitors. Among the different mutations identified T315I is of particular concern since it is not effectively targeted by the majority of Tyrosine Kinase Inhibitors so far available. We developed a novel assay based on peptide nucleic acid (PNA) technology coupled to immunofluorescence microscopy (PNA-FISH) for the specific detection at a single cell level of *BCR-ABL*^*T315I*^ mutation thus improving both, diagnostic resolution and the study of clonal prevalence. Furthermore we developed an additional method based on PNA directed PCR-clamping for the fast and easy detection of the mutation.

**Results:**

The PNA directed PCR clamping allows to detect an amount of mutated template as low as 0.5 %. This method is highly sensitive, specific and cheap and could be applied even in laboratory not equipped for more sophisticated analysis. Furthermore, the PNA FISH method allows to identify a small amount of progenitor cells still present after therapy with specific inhibitors.

**Conclusions:**

We present here two different methods based on PNA for the detection of T315I useful for different purposes. PNA-FISH can be used to study clonal evolution. In addition, this method could help in the study of compound mutations being able to identify two different mutations in a single cell. PNA directed PCR clamping although not superior to sequencing can be applied worldwide even in laboratory not equipped to search for mutations.

## Background

Chronic myeloid leukemia (CML) is characterized by the presence of the Philadelphia chromosome (Ph+) resulting from a translocation between chromosomes 9 and 22 [1]. Ph chromosome gives origin to the *BCR-ABL1* fusion gene coding for a constitutive active tyrosine kinase protein. Despite high response rate to specific tyrosine kinase inhibitors (TKI), primary and secondary resistance have been observed: upfront resistance is defined as lack of initial response and acquired resistance is defined as loss of an established response. *BCR-ABL1* kinase domain (KD) mutations represent a well established cause of resistance to tyrosine kinase inhibitors [2]. Among different mutations identified the frequently observed T315I is of particular concern since it is not effectively targeted by the majority of TKIs so far available [3]. The only drug showing activity against T315I positive CML is ponatinib. [4]

Currently, the recommended method for *BCR-ABL1* mutation detection is the sequencing of the KD [5]. This is time consuming and it allows to reach a maximum sensitivity of 10–15 %.

The latter point represents a limit, as frequently mutated clones may be present at a lower percentage [6].

A relative new technique such the “ultra deep sequencing” allows to reach a very high level of sensitivity but it is far from been routinely applicable in world-wide laboratories [7].

The availability of a simple, sensitive and quick assay, allowing a rapid detection of the T315I mutation is therefore crucial, as the detection of this mutation represents an important element in clinical decision for CML patients.

Peptide Nucleic Acid (PNA) is a potent DNA mimic in terms of sequence specific hybridization. PNA/DNA is thermally more stable than DNA/DNA or DNA/RNA duplexes, [8] but PNA sequences cannot be extended by DNA polymerase [9]. As consequence, PNA/DNA duplex suppresses DNA amplification. Furthermore, PNA/DNA hybridization shows a greater single-base-pair mismatch discrimination than the corresponding DNA/DNA duplex.

**Fig. 1:**
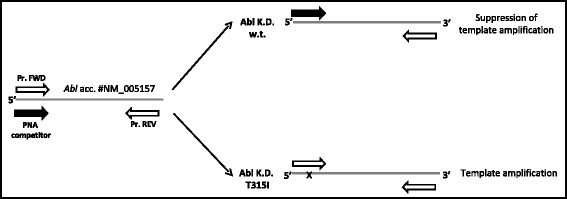
Experimental design: perfect PNA/DNA hybridization occurs when template sequence is w.t., thus leading to suppression of PCR amplification. By contrast, when in presence of single base-pair mismatch (i.e. T315I, indicated by x), PNA/DNA duplex is strongly destabilized allowing template amplification. Empty and filled arrows represent DNA primers used for PCR amplification and PNA competitor, respectively

Based on this premise and previous data [10] we developed a novel and sensitive detection assay in order to quickly and easily identify T315I mutation in CML patients by PNA directed PCR clamping. The experimental design forecasts that both PNA and PCR primer target sites overlap, thus leading to a direct competition towards complementary DNA (Fig. 1). When perfect matching occurs PNA-template hybridization is favoured more than primer template duplex and DNA amplification is suppressed. Conversely, a single mismatch destabilizes the PNA-template duplex, favouring the hybridization between template and primer thus allowing template amplification. Competitor PNA sequence was designed to perfectly match wild-type (WT) template sequence. Therefore, when a single base pair mismatch occurs (like in the case of T315I) PNA-template stability is strongly impaired and DNA amplification favoured.

In addition, in order to identify the presence of *BCR-ABL1 T315I* mutation at the single-cell level, we set up a fluorescently-labelled PNA probe, coupled to FISH technology.

This method allows to distinguish single mutated cells from wild type cells in the context of Ph positive hematopoiesis. Here we report the two strategies (PNA clamping and PNA FISH) for a highly sensitive and very specific detection of *BCR-ABL1 T315I,* applied in the clinical setting of BCR-ABL T315I monitoring.

## Methods

### PNA-PCR clamping for BCR-ABL1 T315I mutation

The study was approved by the local ethic committee of San Luigi Hospital, Orbassano, Turin. After written informed consent BM aspirates were obtained from 17 imatinib resistant CML patient and 1 Ph + Acute Lymphoblastic Leukemia (ALL), all displaying T315I mutation detected by Sanger Sequencing. In addition, as negative control, 25 CML patients without mutations and 15 healthy subjects were examined. Detection of *BCR-ABL1* was performed by capillary Sanger Sequencing method and analyzed by sequencing with BigDye terminator v3.1 (Applied Biosystem, Foster City, California CA) and capillary electrophoresis on ABI PRISM 3130XL (Applied Biosystem, Foster City, California CA). The sensitivity of this method was previously estimated by serial dilutions experiments to be approximately10 %.

Primer Sequences were as follow:

FWD: 5’-tatcatcactgagttcatgacc-3’;

REV: 5’-ggccaaaatcagctaccttcac-3’;

PNA competitor: OO-atatcatcactgagttcat-Lys

Competitor PNA sequence was designed to perfectly match WT template sequence.

PNA-PCR clamping conditions for *BCR-ABL1 T315I* mutation detection were as follow:

1st step PCR: 94 °C 3 min, (94 °C 30 s, 55 °C 30 s, 72 °C 1 min 40 s) for 40 cycles, 72 °C 5 min; 2nd step PCR: 94 °C 3 min, (94 °C 30 s, 65 °C 20 s, 55 °C 50 s, 72 °C 15 s) for 30 cycles. Sensitivity was assessed mixing, at different ratio, mutated (T315I) and WT template. Dilutions were as follow: 100, 20, 10, 5, 1, 0.5 and 0 % T315I mutated *versus* WT template. PCR amplification was carried-out in absence (−) or in presence (+) of competitor PNA, at a concentration 3× greater than primer FWD. The amplification performed without (−) PNA represents an internal positive control displaying the efficiency of template amplification.

### PNA FISH for the detection of BCR-ABL1 T315I mutation

In addition, for the detection of T315I mutation in progenitor cells we set up a method based on fluorescently-labelled PNA probe (PNA FISH). CD34^+^ cells were enriched by magnetic cell sorting (MACS; Miltenyi Biotec, Bergisch Gladbach, Germany) according to the manufacturer’s protocol. PNA probe was designed on the human *BCR-ABL1 T315I* cDNA. The single nucleotide mismatch falls just in the middle of the sequence. The probe has been further tagged by fluorescinated dye at its amino-terminus. The sequence is as follow: Alexa488-OO-TATCATTGAGT-Lys. Detection of *BCR-ABL T315* by PNA FISH was performed as previously described [10]. At least 500 cells have been evaluated for each sample. A positive control is added in each reaction to distinguish between negative results and lack of hybridization.

## Results and discussion

### PNA FISH and PNA direct PCR clamping allow to detect T315I mutation in CML patients

**Fig. 2:**
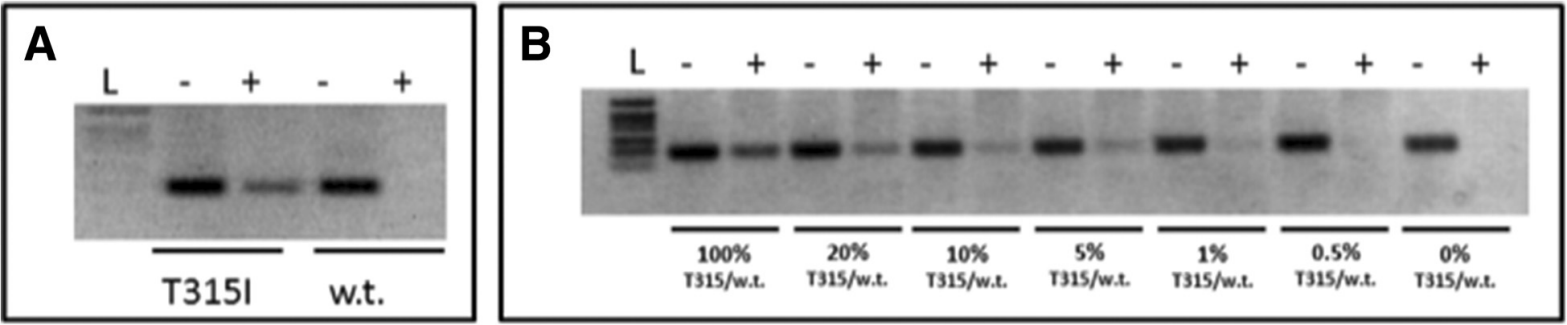
Abl kinase domain T315I point mutation detection analysis by PNA directed PCR clamping. A representative result of the analysis carried out on cDNAs isolated from patients affected by imatinib resistance CML is represented in panel (**a**). PCR amplification was carried-out in absence (−) or in presence (+) of competitor PNA, at a concentration 3× greater than primer FWD. The amplification performed without (−) PNA represents an internal positive control displaying the efficiency of template amplification. As result only when PNA-template duplex stability is weakened because of the mutation an efficient template amplification occurred. L: DNA ladder. Sensitivity was assessed mixing, at different ratio, mutated (T315I) and w.t. template panel (**b**). Dilutions were as follow: 100, 20, 10, 5, 1, 0.5 and 0 % mutated (T315I) *versus* w.t. template

Twelve patients displaying T315I mutations and 15 with *BCR-ABL1* WT were examined either by direct DNA sequencing or by PNA directed PCR clamping displaying identical readout. The results and PCR conditions are summarized in Fig. 2 panel a. As expected, when the affinity between PNA and template is lowered due to the presence of the T315I mutation, DNA template is efficiently amplified. By contrast, in case of WT template the high affinity PNA/template duplex abolished PCR amplification. To test the sensitivity of the method, serial dilutions with WT and mutated T315I templates were performed keeping constant the total template amount. Surprisingly, the method displays a quite high sensitivity, allowing to detect amount of mutated template as low as 0.5 % (Fig. 2, panel b), which are not identified by classical sequencing allowing the identification of T315I mutation even when present at low amount. This method is highly sensitive, specific and cheap and could be applied even in laboratory not equipped for more sophisticated analysis. Five out of 17 CML patients carrying T315I, 1 Ph + ALL and 10 WT CML patients have been tested by PNA FISH.

This technique allowed us to identify T315I mutation at a single cell level. In particular we applied this technique to CD34+ cells to investigate the mutation in the progenitor cell compartment.

**Fig. 3:**
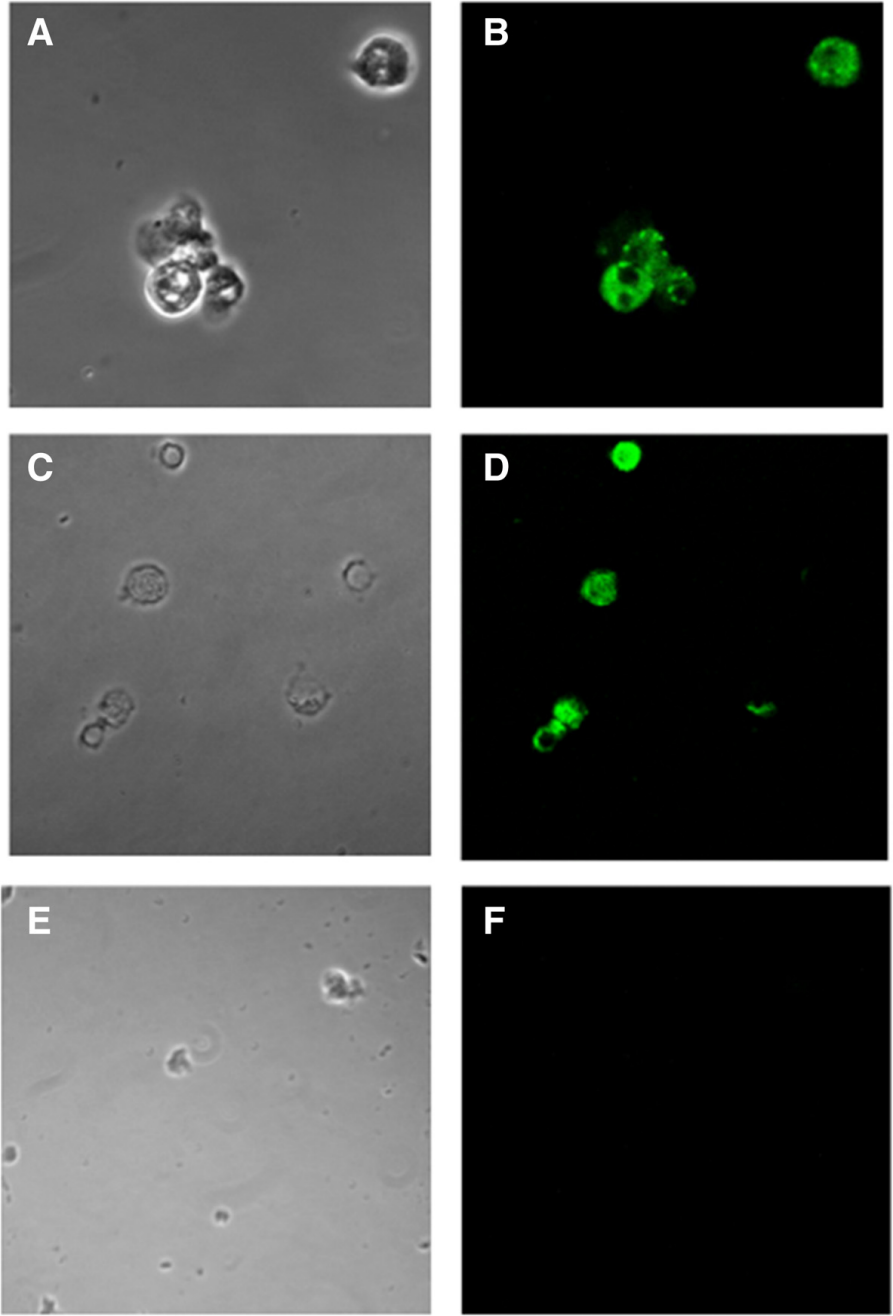
Detection *of BCR-ABL T315I* mutation by PNA. Detection of *BCR-ABL* T315I mutation by PNA (green signal) in CD34+ cells enriched from CML patients carrying T315I mutation. Panel **a** and **b** show T315I positive cells from a patient with 60 % of mutated cells positive cells, panel **c** and **d** show T315I positive cells from a patient with 45 % of mutated cells. Negative control (**e**, **f**) is represented by a CML patient without T315I mutation. No specific PNA green signal can be detected in the absence of the mutation

More in details, we found a residual amount of 3 % of CD34+ positive cells in a patient who became negative for the T315I mutation by Sanger sequencing after ponatinib treatment and acquired T317 mutation at the time of evaluation by PNA. In additional 4 patients positive for T315I mutation by Sanger sequencing, PNA FISH detected the mutation in respectively 20, 35, 45 % (Fig. 3 panel c, d) and 60 % (Fig. 3 panel a, b) of CD34+ cells. Finally in the case of Ph + ALL with increasing values of *BCR-ABL1* transcript during dasatinib treatment and negative for T315I by Sanger sequencing we found about 2 % of cells with T315I mutation by PNA FISH. These cells were undetectable after hematopoietic stem cell transplant, in accordance with a progressive decrease of *BCR-ABL1* transcript.

## Conclusions

We suggest that this approach could be extended to other relevant and frequent *BCR-ABL1* mutations thus allowing to drive clinical decisions after TKI failure. Importantly, it is now known that the emergence of compound mutations in a *BCR-ABL1* allele usually confer ponatinib resistance. More in general, it was shown that *BCR-ABL1* compound mutants confer different levels of TKI resistance [11], thus requiring a rational and patient adapted selection of drugs to optimize the clinical outcome. PNA-FISH allows to identify compound mutation in single cells and to predict response to therapy. Furthermore, PNA-FISH technology allows to provide an answer to many questions including the possibility of the persistence of mutated stem/progenitor cells in patients in MMR and the significance of the presence of small mutated clones at diagnosis. Finally, it allows to follow up clonal evolution during TKI therapy.

## Abbreviations

ALL: Acute lymphoblastic leukemia
CML: Chronic myeloid leukemia
FISH: Fluorescence *in situ* hybridization
KD: Kinase domain
MACS: Magnetic activated cell sorting
MMR: Major molecular remission
PCR: Polymerase chain reaction
Ph: Philadephia
PNA: Peptide nucleic acid
TKI: Tyrosine kinase inhibitors
WT: Wild type
